# Genetic relationship between poultry and wild bird viruses during the highly pathogenic avian influenza H5N6 epidemic in the Netherlands, 2017–2018

**DOI:** 10.1111/tbed.13169

**Published:** 2019-04-05

**Authors:** N. Beerens, R. Heutink, S. Pritz‐Verschuren, E. A. Germeraad, S. A. Bergervoet, F. Harders, A. Bossers, G. Koch

**Affiliations:** ^1^ Wageningen Bioveterinary Research Lelystad the Netherlands

**Keywords:** Avian influenza, full genome sequencing, genetic analysis, H5N6

## Abstract

In the Netherlands, three commercial poultry farms and two hobby holdings were infected with highly pathogenic avian influenza (HPAI) H5N6 virus in the winter of 2017–2018. This H5N6 virus is a reassortant of HPAI H5N8 clade 2.3.4.4 group B viruses detected in Eurasia in 2016. H5N6 viruses were also detected in several dead wild birds during the winter. However, wild bird mortality was limited compared to the caused by the H5N8 group B virus in 2016–2017. H5N6 virus was not detected in wild birds after March, but in late summer infected wild birds were found again. In this study, the complete genome sequences of poultry and wild bird viruses were determined to study their genetic relationship. Genetic analysis showed that the outbreaks in poultry were not the result of farm‐to‐farm transmissions, but rather resulted from separate introductions from wild birds. Wild birds infected with viruses related to the first outbreak in poultry were found at short distances from the farm, within a short time frame. However, no wild bird viruses related to outbreaks 2 and 3 were detected. The H5N6 virus isolated in summer shares a common ancestor with the virus detected in outbreak 1. This suggests long‐term circulation of H5N6 virus in the local wild bird population. In addition, the pathogenicity of H5N6 virus in ducks was determined, and compared to that of H5N8 viruses detected in 2014 and 2016. A similar high pathogenicity was measured for H5N6 and H5N8 group B viruses, suggesting that biological or ecological factors in the wild bird population may have affected the mortality rates during the H5N6 epidemic. These observations suggest different infection dynamics for the H5N6 and H5N8 group B viruses in the wild bird population.

## 1 INTRODUCTION

Highly pathogenic avian influenza (HPAI) H5 viruses related to the H5N1 virus A/Goose/Guangdong/1/1996 (Xu, Subbarao, Cox, & Guo, 1999) caused outbreaks in Asia, Europe, Africa and North America. The transcontinental spread of these viruses has been linked to dissemination by migratory wild birds (Global Consortium for H5N8, 2016). The H5N1 viruses have evolved into novel reassortant HPAI H5 viruses of different neuraminidase (NA) subtypes (Lee, Bertran, Kwon, & Swayne, 2017). Of these reassortant viruses, the clade 2.3.4.4 H5 viruses further evolved into four genetic groups, named A to D (Lee et al., 2016). Outbreaks in poultry in the Netherlands were caused by H5N8 group A viruses in 2014 (Bouwstra, Heutink, et al., 2015; Bouwstra, Koch, et al., 2015), and H5N8 group B viruses in 2016 (Beerens et al., 2017). In December 2017, an outbreak of HPAI H5N6 was detected on a commercial poultry farm in the Netherlands. We recently showed that this virus is a novel reassortant of the H5N8 clade 2.3.4.4 group B viruses (Beerens et al., 2018), which were first detected at the Russia–Mongolia border in May 2016. The H5N6 virus obtained novel polymerase basic 2 (PB2) and NA segments derived from Eurasian low pathogenic avian influenza (LPAI) viruses. After the first outbreak in poultry, H5N6 viruses were detected on two additional commercial poultry farms and two hobby holdings between December 2017 and March 2018. In this period, H5N6 virus was also detected in several dead wild birds that were tested in the passive wild bird surveillance programme in the Netherlands. Infected wild birds were also found in several other European countries (Germany, United Kingdom, Ireland, Denmark, Sweden, Finland and Slovakia). Wild bird mortality caused by H5N6 viruses was limited compared to that observed during the H5N8 epidemic in 2016–2017. However in 2014, no dead wild birds infected with the H5N8 group A virus were detected. These observations suggest striking differences in the pathogenicity of these HPAI H5 viruses in wild birds.

After March 2018, the virus was not detected in wild birds or poultry in the Netherlands for several months. However, in August two dead wild birds infected with H5N6 virus were found again. Detection of the virus in late summer suggests long‐term circulation of H5N6 viruses in the local wild bird population. Alternatively, it may be a novel incursion of H5N6 virus due to the start of fall migration of wild birds to Europe. In this study, we analysed the genetic relationship between the viruses isolated from poultry and wild birds. This analysis will provide insight in whether the farms were infected by farm‐to‐farm transmission, or by separate introductions from wild birds. In addition, the analysis will provide insight in the origin of the H5N6 virus that was detected in a wild bird in late summer. Finally, the pathogenicity of the H5N6 virus in ducks was compared to that of the H5N8 viruses that caused epidemics in 2014 and 2016. These combined results will provide more insight in the HPAI H5N6 epidemic, which affected both wild birds and poultry in the Netherlands in 2017–2018.

## 2 METHODS

### 2.1 Virus detection and subtyping

Viral RNA was extracted from tracheal or cloacal swabs from dead wild birds using the MagNa Pure 96 (Roche). For commercial poultry farms, pools of five samples from clinically affected chickens or ducks were used. Tracheal and cloacal swabs were pooled separately. The samples were tested in a matrix gene real‐time PCR, which detects all avian influenza (AI) virus subtypes, as described previously (Bouwstra, Heutink, et al., 2015). The positive samples were then subtyped using a H5‐specific real‐time PCR (Slomka et al., 2007), as recommended by the European Union reference laboratory. The sequence of the hemagglutinin (HA) cleavage site and the NA subtype was determined by Sanger sequencing (Beerens et al., 2018).

### 2.2 Complete genome sequencing and analysis

Viral RNA was purified using the High Pure Viral RNA kit (Roche), and amplified using universal eight‐segment primers and directly sequenced, as described previously (Bouwstra, Heutink, et al., 2015). Briefly, purified amplicons were sequenced at high coverage (average >1000 per nucleotide position) using the Nextera library preparation method and Illumina MiSeq paired‐end 150 base pairs sequencing. Quality control‐passed sequence reads were mapped using the ViralProfiler‐Workflow, an extension of the CLC Genomics Workbench (Qiagen, Germany), as previously described (Beerens et al., 2017). The consensus sequences generated in this study were submitted to the GISAID database. The GISAID accession numbers are listed in Table 1.

**Table 1:** GISAID accession numbers of the sequences generated or used in this study

Isolate ID	Isolate name	Host	Location	Collection date	Code
Poultry farms					
EPI_ISL_287907^a^	A/duck/Netherlands/17017237‐001‐005/2017	Duck	Biddinghuizen	7‐12‐2017	BH
EPI_ISL_332430	A/chicken/Netherlands/18003041‐001‐005/2018	Chicken	Oldekerk	25‐2‐2018	OK
EPI_ISL_332431	A/duck/Netherlands/18003885‐001‐005/2018	Duck	Kamperveen	13‐3‐2018	KV
Hobby holdings					
EPI_ISL_332434	A/peacock/Netherlands/17017775‐064‐068/2017	Peacock	Biddinghuizen	15‐12‐2017	HBH1
EPI_ISL_332432	A/guineafowl/Netherlands/17017775‐020‐024/2017	Guineafowl	Biddinghuizen	15‐12‐2017	HBH2
EPI_ISL_332433	A/bird/Netherlands/17017775‐035‐039/2017	Bird	Biddinghuizen	15‐12‐2017	HBH3
EPI_ISL_332435	A/chicken/Netherlands/18000887‐005/2018	Chicken	Rhoon	20‐1‐2018	HR1
EPI_ISL_332436	A/peacock/Netherlands/18000887‐006/2018	Peacock	Rhoon	20‐1‐2018	HR2
EPI_ISL_332437	A/turkey/Netherlands/18000887‐007‐010/2018	Turkey	Rhoon	20‐1‐2018	HR3
Wild birds					
EPI_ISL_288410^a^	A/mute swan/Netherlands/17017377‐001/2017	Mute swan	Elburg	9‐12‐2017	wb1
EPI_ISL_288412^a^	A/tufted duck/Netherlands/17017367‐007/2017	Tufted duck	Hulshorst	9‐12‐2017	wb2
EPI_ISL_288409^a^	A/mute swan/Netherlands/17017367‐012/2017	Mute swan	Hulshorst	9‐12‐2017	wb3
EPI_ISL_332438	A/mute swan/Netherlands/17017903‐002/2017	Mute swan	Elburg	19‐12‐2017	wb5
EPI_ISL_332439	A/peregrine falcon/Netherlands/18003274‐001/2018	Peregrine falcon	Westernieland	1‐3‐2018	wb7
EPI_ISL_332440	A/buzzard/Netherlands/18004242‐002/2018	Buzzard	Zandfoort	15‐3‐2018	wb9
EPI_ISL_332441	A/mallard/Netherlands/18012508‐017/2018	Mallard	Eemmeer	24‐8‐2018	wb10

Other European sequences^b^						Originating Lab	Authors
EPI_ISL_289714	A/black‐headed gull/Netherlands/29/2017	Black‐headed gull	Netherlands	18‐12‐2017	NL1	Erasmus Medical Center	Poen MJ et al.
EPI_ISL_302823	A/great black‐backed gull/Netherlands/1/2018	Great black‐backed gull	Netherlands	23‐1‐2018	NL2	Erasmus Medical Center	Poen MJ et al.
EPI_ISL_302824	A/eurasian wigeon/Netherlands/1/2018	Eurasian wigeon	Netherlands	7‐2‐2018	NL3	Erasmus Medical Center	Poen MJ et al.
EPI_ISL_292223	A/mute_swan/England/AVP_18_001986/2017	Mute swan	United Kingdom	31‐12‐2017	UK1	Animal and Plant Health Agency	Seekings J. et al.
EPI_ISL_292225	A/canada_goose/England/AV58_18OPpoolEP1/2018	Canada goose	United Kingdom	5‐1‐2018	UK2	Animal and Plant Health Agency	Seekings J. et al.
EPI_ISL_292224	A/pochard_duck/England/AVP_18_003254/2018	Pochard duck	United Kingdom	10‐1‐2018	UK3	Animal and Plant Health Agency	Seekings J. et al.
EPI_ISL_291109	A/common_pochard/Germany‐BY/AR09‐18‐L02421/2017	Common pochard	Germany	28‐12‐2018	DE1	Friedrich‐Loeffler‐Institut	N/A

BH, Biddinghuizen; OK, Oldekerk; KV, Kamperveen; Hobby holdings: HBH, hobbyfarm Biddinghuizen; HR, hobbyfarm Rhoon; wb1–9; wild birds 1–9.

a Sequences generated in a previous study, see reference (8).

b We acknowledge the authors, originating and submitting laboratories of the sequences from GISAID's EpiFlu™ Database on which this research is based. All submitters of data may be contacted directly via the GISAID website http://www.gisaid.org.

### 2.3 Phylogenetic network

The eight‐gene‐segment alignments were manually concatenated to generate a single alignment that was used to construct phylogenetic networks using the median‐joining method implemented in the program NETWORK, as described previously (Bataille, van der Meer, Stegeman, & Koch, 2011). This model‐free method uses a parsimony approach based on pairwise differences to connect each sequence to its closest neighbour, and allows creation of internal nodes (‘median vectors’), which could be interpreted as unsampled or extinct ancestral genotypes to link the existing genotypes in the most parsimonious way (Bandelt, Yao, Bravi, Salas, & Kivisild, 2009). In the analysis, we also included sequences from three wild bird viruses that were isolated in research programmes in the Netherlands (NL1‐3); however, a forth sequence was excluded because of a reassortment of the polymerase acidic (PA)gene segment. The sequences of publically available Dutch and European sequences that were used in this study are listed in Table 1.

### 2.4 IVPI in ducks

The Intravenous Pathogenicity Index (IVPI) was determined for viruses H5N6 2017 (A/duck/Netherlands/17017237‐011‐015/2017, EPI_ISL_287907), H5N8 2016 (A/duck/Netherlands/16014829‐001005/2016, EPI_ISL_268629) and H5N8 2014 (A/chicken/Netherlands/14015531/2014, EPI_ISL_168075) using Pekin ducks, according to the standard procedure (OIE, 2015). Ten 6‐week‐old Pekin ducks were purchased from a commercial breeder. The animals were tested for the absence of antibodies against AI using ELISA (Influenza Ab Test kit, IDEXX Laboratories). After intravenous inoculation, the ducks were monitored for clinical signs and mortality for 10 days. The experiment was performed in biosecurity level‐3 facilities, under the approval of the Central Animal Experiments Committee (licence number 2017.D‐0054.001) in the Netherlands.

## 3 RESULTS

### 3.1 Detection of HPAI H5N6 in poultry and wild birds

**Figure 1:**
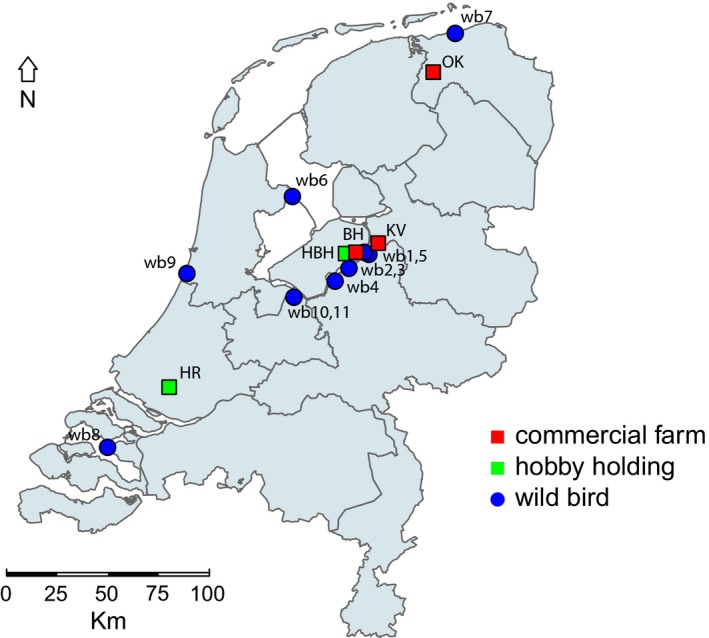
Map of the Netherlands, showing the location of commercial poultry farms (red), hobby holdings (green) and dead wild birds infected with HPAI H5N6 virus (blue). Commercial poultry farms: BH, Biddinghuizen; OK, Oldekerk; KV, Kamperveen; Hobby holdings: HBH, hobbyfarm Biddinghuizen; HR, hobbyfarm Rhoon; wild birds, wb 1–9

The first introduction of HPAI H5N6 into a commercial poultry holding was detected on an indoor farm with meat ducks on 7 December 2017, in the municipality of Biddinghuizen. The farm was located near a lake in the central part of the Netherlands, were large quantities of wild waterfowl are present. The second outbreak of H5N6 virus occurred in a parent breeder flock in the municipality of Oldekerk, on 25 February 2018. The farm was situated in a water‐rich area, near a channel, with an abundant presence of wild waterfowl. The third outbreak was detected on an indoor farm with meat ducks in the municipality of Kamperveen. This farm was also located in the central lake area of the Netherlands. In addition to these outbreaks in commercial poultry holdings, two hobby holdings became infected with H5N6 viruses. A hobby holding in Biddinghuizen tested positive for HPAI H5N6 viruses on 15 December 2017, and in Rhoon on 20 January 2018. The geographical locations of the commercial poultry holdings (shown in red) and hobby holdings (shown in green) infected with H5N6 virus are depicted in Figure 1.

In the passive wild bird surveillance programme, diagnostic testing for AI is performed for wild birds found dead. Between 1 November 2017 and 1 April 2018, 281 dead birds were tested, of which 13 birds tested positive for HPAI H5N6 (Table 2). Early in the epidemic, mostly birds of the family *Anatidae* (ducks, geese and swans) were affected, whereas later also birds of prey tested positive for the virus. The virus was first detected in a mute swan found dead in the central lake area of the Netherlands, on 9 December 2017. After the detection of the virus in a common buzzard on 15 March 2018, the virus was not detected in wild birds for several months. However, a mallard found dead on 24 August 2018 in the central lake area of the Netherlands tested positive again for H5N6 (Table 2). A few days later, the virus was also detected in a Eurasian marsh harrier found dead in the same area. The geographical locations where H5N6‐infected dead wild birds were found are depicted in Figure 1 (shown in blue).

**Table 2:** HPAI H5N6 virus‐infected wild birds detected by passive wild bird surveillance

Location	Date	Family	Species	#^a^	Code
Elburg	9‐12‐2017	Anatidae	Mute swan ‐ *Cygnus olor*	1	wb1
Hulshorst	9‐12‐2017	Anatidae	Tufted duck ‐*Aythya fuligula*	1	wb2
Hulshorst	9‐12‐2017	Anatidae	Mute swan ‐ *Cygnus olor*	5	wb3
Harderwijk	14‐12‐2017	Anatidae	Mute swan ‐ *Cygnus olor*	1	wb4
Elburg	19‐12‐2017	Anatidae	Mute swan ‐ *Cygnus olor*	1	wb5
Enkhuizen	21‐02‐2018	Anatidae	Greater scaup ‐ *Aythya marila*	1	wb6
Westernieland	1‐03‐2018	Falconidae	Peregrine falcon ‐ *Falco peregrinus*	1	wb7
Stavernisse	14‐03‐2018	Accipitridae	Buzzard ‐ *Buteo buteo*	1	wb8
Zandfoort	15‐03‐2018	Accipitridae	Buzzard ‐ *Buteo buteo*	1	wb9
Eemmeer	24‐08‐2018	Anatidae	Mallard ‐ *Anas platyrhynchos*	1	wb10
Eemmeer	31‐08‐2018	Accipitridae	Eurasian marsh harrier ‐ *Circus aeruginosus*	1	wb11

wb1–9; wild birds 1–9.

a Number of birds found dead.

### 3.2 Genetic analysis of the H5N6 epidemic

**Figure 2:**
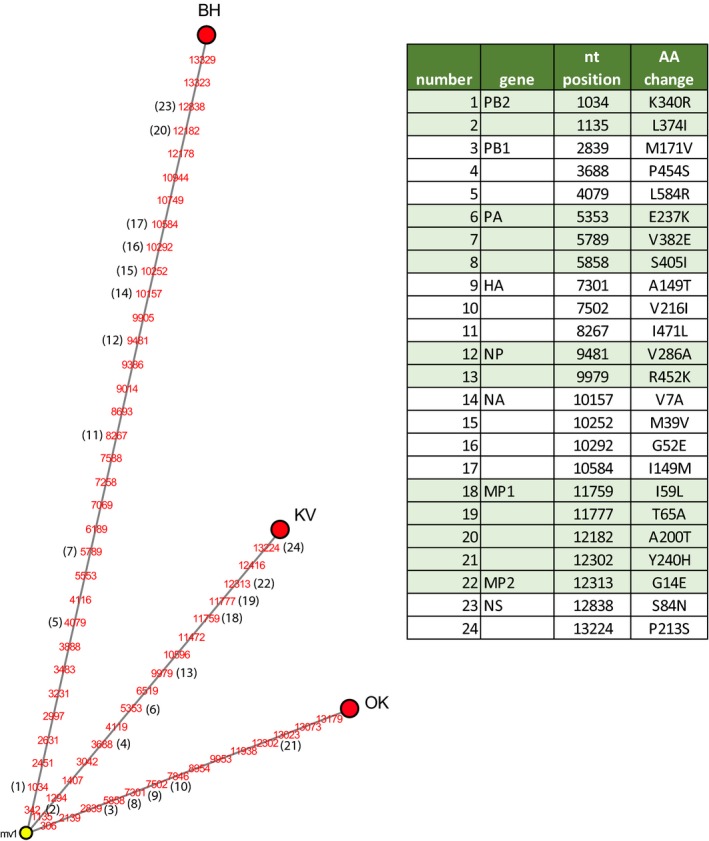
Median‐joining network analysis of HPAI H5N6 viruses isolated from three commercial farms in the Netherlands. The median‐joining network was generated for the full genome sequence of the viruses. The full genome sequence was obtained by combining the sequences from the eight individual gene segments. This network includes all the most parsimonious trees linking the three sequences. Each sequence genotype is represented by a red circle; the median vector is represented by a yellow circle. The numbers (red) refer to nucleotide (nt) positions that are different between the sequences. A number between brackets (black) marks the mutations resulting in amino acid (AA) change. The specific AA changes are listed in the table. BH, Biddinghuizen; OK, Oldekerk; KV, Kamperveen

We previously determined the complete genome sequences of HPAI H5N6 viruses isolated from the first outbreak on the commercial poultry in Biddinghuizen and three wild birds (wb 1–3, Table 2) found dead nearby this farm (Beerens et al., 2018). In the current study, we performed deep sequencing to determine the complete genome sequences of H5N6 viruses that were detected later in 2017–2018 in commercial and hobby poultry holdings, and in dead wild birds. To analyse the genetic relationship between viruses isolated from commercial poultry, a median‐joining network analysis was performed (Figure 2). We identified 47 nucleotide differences between the genomes of the viruses detected at the farm in Biddinghuizen (outbreak 1) and Oldekerk (outbreak 2), resulting in 16 amino acid changes (Figure 2). The genomes of the viruses detected at the farms in Oldekerk and Kamperveen (outbreak 3) differed by 30 nucleotides, and 13 amino acids. The large number of genetic differences between the viruses at the poultry farms suggests that the infections are not caused by virus transmission between the farms. Therefore, likely the farms were infected by separate introductions from wild birds.

**Figure 3:**
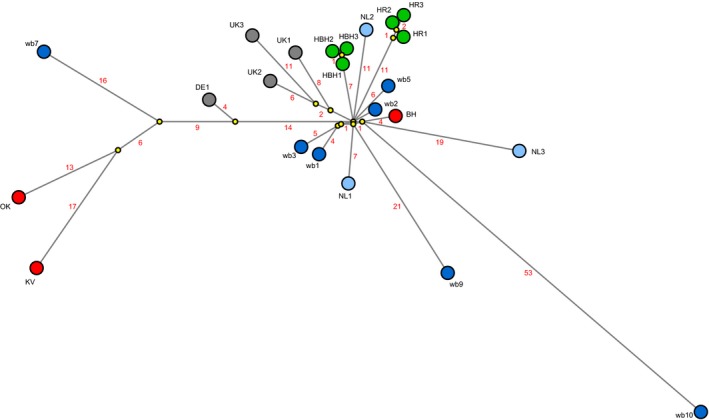
Median‐joining network showing the genetic relationship between HPAI H5N6 viruses isolated from commercial poultry farms (red), hobby holdings (green) and from dead wild birds tested in the passive surveillance programme (dark blue) in the Netherlands. Also shown are viruses isolated from wild birds in other (research) programmes in the Netherlands (light blue), and other European countries (grey). Predicted median vectors are shown in yellow. The virus isolates used for this analysis are listed in Table 1, which also provides the GISAID accession numbers

The genetic relationship between H5N6 viruses isolated from poultry and wild birds was also studied. In this network analysis, we included publically available sequences of other European H5N6 virus isolates (Table 1). The analysis identified several wild bird viruses related to the virus isolated from the farm in Biddinghuizen (Figure 3). The genetically most closely related wild bird virus (wb 2) contained eight nucleotide differences, and was isolated from a tufted duck. This dead bird was found 2 days after detection of the virus at the farm (distance 9 km) (Table 3). Two mute swans (wb 1 and 3) that were found dead in the central lake area near Biddinghuizen on the same day also carried highly similar viruses. For the two hobby holding, viruses isolated from three different poultry species were sequenced (Table 1). These sequences differed by one or two nucleotides within one holding (Figure 3, HBH1–3 and HR1–3). The viruses isolated from the hobby holdings are also found in the cluster of viruses related to the commercial poultry farm in Biddinghuizen. This cluster also contains viruses detected later in winter 2017–2018, at longer distances from the farm (Table 3). This may suggest that the H5N6 virus first spread locally in the central lake area of the Netherlands, and later spread to other regions of the Netherlands and the United Kingdom. No wild bird viruses directly related to the viruses detected on the commercial poultry farms in Oldekerk and Kamperveen were detected. This suggests that more viruses were introduced in the Netherlands than have been detected in the passive wild bird surveillance programme.

**Table 3:** Time, distance and genetic differences between first and later detections of HPAI H5N6 virus

Location	Host	Date	Days^a^	km^b^	nt^c^	Code
Poultry farms						
Biddinghuizen	Duck	7‐12‐2017	‐	‐	‐	BH
Oldekerk	Chicken	25‐2‐2018	80	97	47	OK
Kamperveen	Duck	13‐3‐2018	96	12	49	KV
Hobby holdings						
Biddinghuizen	PEACOCK	15‐12‐2017	8	5	12	HBH1
Biddinghuizen	Guineafowl	15‐12‐2017	8	5	14	HBH2
Biddinghuizen	Bird	15‐12‐2017	8	5	14	HBH3
Rhoon	Chicken	20‐1‐2018	44	114	17	HR1
Rhoon	Peacock	20‐1‐2018	44	114	18	HR2
Rhoon	Turkey	20‐1‐2018	44	114	19	HR3
Wild birds						
Elburg	Mute swan	9‐12‐2017	2	6	11	wb1
Hulshorst	Tufted duck	9‐12‐2017	2	9	8	wb2
Hulshorst	Mute swan	9‐12‐2017	2	9	12	wb3
Elburg	Mute swan	19‐12‐2017	12	4	11	wb5
Westernieland	Peregrine falcon	1‐3‐2018	84	118	42	wb7
Zandfoort	Buzzard	15‐3‐2018	98	84	26	wb9
Eemmeer	Mallard	24‐08‐2018	260	35	57	wb10

BH, Biddinghuizen; OK, Oldekerk; KV, Kamperveen; Hobby holdings: HBH, hobbyfarm Biddinghuizen; HR, hobbyfarm Rhoon; wb1–9; wild birds 1–9.

a Days after first outbreak in Biddinghuizen.

b Distance in kilometres from the poultry farm in Biddinghuizen.

c Number of nucleotide differences with the virus detected in Biddinghuizen.

The network analysis shows that the virus detected in a wild bird late in August 2018 shares a common ancestor with the cluster of viruses related to the first outbreak in Biddinghuizen. This virus differs at 53 nucleotide positions from the predicted common ancestor, and 57 nucleotides from the virus detected in outbreak 1 in December 2017. These changes may have been introduced by ongoing virus evolution in the 9 months between these two detections. These results suggest that the wild bird virus detected in late August most likely evolved from the viruses detected earlier in the Netherlands in December 2017.

### 3.3 Pathogenicity of the H5N6 virus

**Figure 4:**
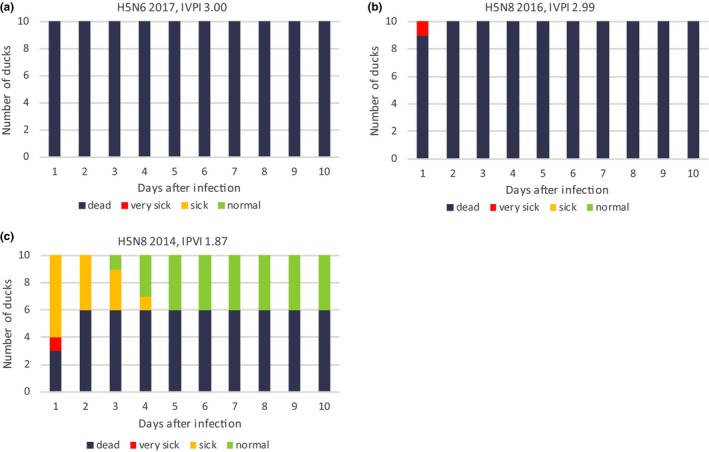
The intravenous pathogenicity index (IVPI) scores in ducks. The results of the experiment for (a) H5N6 2017, (b) H5N8 2016 and (c) H5N8 2014 are shown. Ten 6‐week‐old Pekin ducks were inoculated and monitored for clinical signs for 10 days. For every day the number of normal (green), sick (orange), very sick (red) and dead (dark grey) ducks is shown

The pathogenicity of the H5N6 virus was determined in Pekin ducks, and compared to that of the H5N8 viruses detected in the Netherlands in 2014 and 2016. For this experiment, the viruses isolated from the index cases in poultry were used. We infected 10 ducks of 6 weeks old by intravenous injection of the virus, and monitored them for 10 days for clinical signs and mortality. The experiment was performed according to the standard protocol for determining the intravenous pathogenicity index (IVPI). After infection with the H5N6 virus from 2017 all 10 ducks died on the first day. For the H5N8 2016 virus, nine ducks died on day 1, and one duck on day 2. This resulted in an IVPI score of 3.0 for both viruses (Figure 4a,b). For the H5N8 2014 virus, four ducks survived the 10 days of the experiment, resulting in an IVPI score of 1.9 (Figure 4c). The reduced pathogenicity index of the H5N8 2014 virus correlates with the absence of wild bird mortality during this epidemic. Surprisingly, the IVPI scores of H5N6 and H5N8 2016 viruses measured in ducks were equal, whereas only for H5N8 massive wild bird mortality was observed during the epidemic in 2016–2017.

## 4 DISCUSSION

In December 2017, the Netherlands was first to report HPAI H5N6 in Europe (Beerens et al., 2018). Later in 2017–2018, the H5N6 virus was also detected in wild birds in several other European countries. In the Netherlands, three commercial poultry farms and two hobby holdings were infected with H5N6 viruses. H5N6 viruses were detected in 15 dead wild birds tested in the passive wild bird surveillance programme. We previously showed that this virus is a novel reassortant of the HPAI H5N8 clade 2.3.4.4 group B virus that obtained new PB2 and NA segments derived from Eurasian LPAI viruses (Beerens et al., 2018). Phylogenetic analysis demonstrated that the virus is not related to the H5N6 virus detected in Greece in February 2017, as it carries genetically different PA and PB2 gene segments. The virus is also distinct from the zoonotic Asian H5N6 strains that caused infections in humans. There are currently no human cases associated with this novel H5N6 reassortant virus.

In this study, the complete genome sequences of the H5N6 viruses isolated in the Netherlands were determined to study their genetic relationship. The genomes of the viruses isolated from the three commercial poultry farms differed at a large number of nucleotide positions, resulting in several amino acid changes. This demonstrates that the viruses on the three farms were genetically not closely related, and infection likely resulted from separate introductions from wild birds. This is further supported by the epidemiological investigation, which revealed no links between the poultry farms. Directly after testing positive for H5 virus, poultry was culled and restriction and protection zones were established. These measures were effective to prevent spread of the virus. The results emphasize the need for poultry farmers to prevent direct or indirect contact between poultry and wild birds by strictly following hygienic measures and biosecurity protocols. The route of introduction of the virus into the three indoor poultry houses was not identified, but among others, contaminated persons, materials or vectors (e.g. rodents, insects) may have introduced the virus into the farm.

Wild bird mortality caused by HPAI H5N6 was limited in the Netherlands. The virus was detected in dead wild birds tested in the ongoing passive surveillance programme only after infection of the first poultry farm. Increased public awareness likely contributed to the submission of dead wild birds at that time. In the period November 2017–April 2018, we tested 281 wild birds in the passive surveillance programme, and detected HPAI H5N6 in 13 dead birds found on seven different locations in the Netherlands. Thus, the prevalence of H5N6 viruses among dead wild birds in the winter of 2017–2018 was 4.6%. Wild bird viruses genetically similar to the virus detected in outbreak 1 were detected in the passive wild bird surveillance programme. Similar wild bird viruses were first detected in the central lake area, near outbreak 1, but later this virus was also detected in other regions of the Netherlands and abroad. However, no wild bird viruses genetically related to outbreaks 2 and 3 were isolated. Late in summer, two wild birds found dead in the central lake area of the Netherlands tested positive for HPAI H5N6. Genetic analysis showed that this “late” virus shares a common ancestor with the virus detected in outbreak 1, although these viruses differ at 57 nucleotide positions. As this virus was detected almost 9 months after outbreak 1, this suggests a nucleotide substitution rate of around 5.7 × 10^−3^ substitutions/site/year. This calculated substitution rate is within the range previously estimated for HPAI H5 viruses (Fourment & Holmes, 2015; Rejmanek, Hosseini, Mazet, Daszak, & Goldstein, 2015). This suggests that the virus most likely evolved from the viruses detected earlier in the Netherlands. Fall migration of wild birds from breeding grounds in Siberia to Europe may have started in August, and a novel introduction of H5N6 virus thus cannot be excluded. However, due to high temperatures in summer, it is unlikely that the H5N6 virus persisted in the environment until August (Sooryanarain & Elankumaran, 2015). Therefore, most likely the H5N6 virus persisted in the local wild bird population in the Netherlands, remaining undetected until August. Other European countries (Denmark, Germany) also reported H5N6‐infected birds late in summer again, suggesting that the virus was also still circulating in these Northern European countries. However, sequence analysis will have to be performed to confirm this.

The H5N6 virus appeared less pathogenic in wild birds compared to the H5N8 group B virus, which caused massive wild bird mortality in 2016–2017. However for the H5N8 2014 group A virus, no mortality was observed in the wild bird population. We previously showed that the pathogenicity of the three viruses for galliform birds was similar, for all viruses an IVPI score of 3.0 was measured (Beerens et al., 2017, 2018; Bouwstra, Heutink, et al., 2015). In this study, we performed an IVPI experiment using ducks. We show that the H5N8 2014 virus is less pathogenic in ducks, as a score of 1.9 was measured. Surprisingly, for both group B viruses an IVPI score of 3.0 was measured in ducks. The virus was inoculated intravenously in this experiment, whereas natural infection occurs via the respiratory route. Previous studies showed that the observed pathogenicity of HPAI H5N1 was independent of the route of inoculation (Pantin‐Jackwood, Swayne, Smith, & Shepherd, 2013). However, potential differences in virus attachment and entry via the respiratory tract are not assessed in this experiment. Sequence analysis shows that the HA proteins of the two group B viruses differ at only four positions (results not shown). For these positions, no functional role has been described, which suggests a similar entry efficiency for both viruses. The H5N8 group A and group B viruses differ at numerous amino acid positions in the HA gene (Beerens et al., 2017).

The IVPI experiment was performed using Pekin ducks, which is a domestic duck breed derived from the mallard. Therefore, we expect a similar pathogenicity in the wild mallard, although this may differ for other species of water birds (van den Brand et al., 2018). The fact that no difference in pathogenicity was measured for the two group B viruses in ducks may suggest that biological or ecological factors in the wild bird population have resulted in the reduced mortality observed during the H5N6 epidemic. H5N6 viruses may have been less prevalent than H5N8 viruses in the wild bird population, or the exposure to H5N8 viruses in the previous year may have resulted in immunological protection against infection with H5N6 virus. Furthermore, early in the H5N8 2016 epidemic, the virus was mainly detected in migratory water birds (tufted ducks and Eurasian wigeons), whereas later in the epidemic also birds of prey were infected (Kleyheeg et al., 2017). These predators were most likely infected by feeding on infected preys. During the H5N6 epidemic, the virus was mainly detected in residential birds (mute swans), and later again in birds of prey. Furthermore, genetic analysis provided indications for long‐term circulation of H5N6 viruses in the local wild bird population. These observations suggest different infection dynamics for the H5N6 and H5N8 group B viruses in the wild bird population in the Netherlands.

## CONFLICT OF INTEREST

The authors declare no conflict of interest.
